# Is apparent fixational drift in eye-tracking data due to filters or eyeball rotation?

**DOI:** 10.3758/s13428-020-01414-3

**Published:** 2020-07-23

**Authors:** Diederick C. Niehorster, Raimondas Zemblys, Kenneth Holmqvist

**Affiliations:** 1 Lund University Humanities Lab and Department of Psychology, Lund University, Lund, Sweden; 2 Šiauliai University, Šiauliai, Lithuania; 3 Institute of Psychology, Nicolaus Copernicus University in Torun, Torun, Poland; 4 Department of Psychology, Regensburg University, Regensburg, Germany; 5 Department of Computer Science and Informatics, University of the Free State, Bloemfontein, South Africa

**Keywords:** Eye tracking, Fixational eye movements, Drift, Power spectrum, Signal color

## Abstract

Eye trackers are sometimes used to study the miniature eye movements such as drift that occur while observers fixate a static location on a screen. Specifically, analysis of such eye-tracking data can be performed by examining the temporal spectrum composition of the recorded gaze position signal, allowing to assess its color. However, not only rotations of the eyeball but also filters in the eye tracker may affect the signal’s spectral color. Here, we therefore ask whether colored, as opposed to white, signal dynamics in eye-tracking recordings reflect fixational eye movements, or whether they are instead largely due to filters. We recorded gaze position data with five eye trackers from four pairs of human eyes performing fixation sequences, and also from artificial eyes. We examined the spectral color of the gaze position signals produced by the eye trackers, both with their filters switched on, and for unfiltered data. We found that while filtered data recorded from both human and artificial eyes were colored for all eye trackers, for most eye trackers the signal was white when examining both unfiltered human and unfiltered artificial eye data. These results suggest that color in the eye-movement recordings was due to filters for all eye trackers except the most precise eye tracker where it may partly reflect fixational eye movements. As such, researchers studying fixational eye movements should be careful to examine the properties of the filters in their eye tracker to ensure they are studying eyeball rotation and not filter properties.

## Introduction

When humans fixate a static object to stabilize its retinal image (see Hessels et al., 2018), their eyes still make small movements (Ratliff & Riggs, 1950; Ditchburn & Ginsborg, 1953; Collewijn & Kowler, 2008)—termed fixational eye movements. These consist of microsaccades, fixational drift, and tremor (Martinez-Conde et al., 2004; Rolfs, 2009; Rucci & Poletti, 2015). In recent years, the study of these fixational eye movements has elucidated their myriad functional and perceptual consequences (e.g., Ditchburn et al., 1959; Kuang et al., 2012; Rucci et al., 2018; Engbert 2006; Martinez-Conde et al., 2013).

**Fig. 1:**
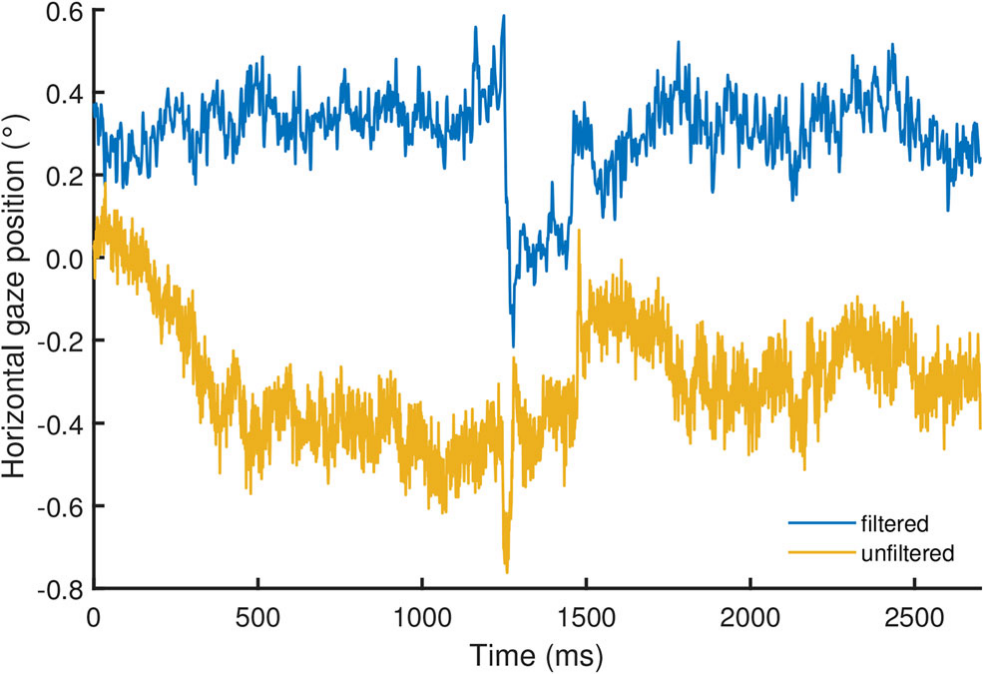
Gaze position data example. Example segments showing gaze position signals recorded at 1000 Hz during two fixations from two different participants with an SR EyeLink 1000 Plus, with its filters either turned on or turned off. The sudden changes in gaze position at 1250 ms and 1500 ms in both signals are likely microsaccades, whereas drift is visible throughout most of the rest of the signals

Figure 1 shows two example segments of eye-tracking data during fixations. In this figure, the recorded gaze position signal during the fixations appears unstable despite the participant attempting to keep their gaze stable at a certain location on the screen. In eye-movement data, these fluctuations are thought to arise from at least two sources, 1) the measurement device, and 2) rotations of the eyeball itself—the fixational eye movements. In this paper, we will refer to these two signal components as *measurement noise* and *fixational eye movements*, respectively. While the fixational eye move ments are of interest to some researchers, the measurement noise originating from the eye tracker is a potential problem, as it may obscure eye movements of interest.

Researchers interested in fixational eye movements such as microsaccades and drift should ensure that the magnitude of measurement noise in their eye-tracker’s output is sufficiently low so as to not obscure these eye movements (Ko et al., 2016). For this reason, such research is usually conducted with the video-based eye trackers that provide the lowest noise levels in their class such as the various SR-Research EyeLinks (e.g., Engbert & Mergenthaler, 2006; Scholes et al., 2015; Nyström et al., 2017) and recently the Tobii Spectrum (Nyström et al., in press). Note also that it has recently been questioned whether video-based eye trackers are suitable for microsaccade research (Holmqvist & Blignaut, 2020). Other researchers interested in fixational eye movements use different measurement techniques that provide even better data quality (lower measurement noise levels), such as Dual-Purkinje eye trackers (Kuang et al., 2012; Horowitz et al., 2007), scleral search coils (McCamy et al., 2015; Ko et al., 2016) and various scanning laser opthalmoscopes (Stevenson et al., 2010; Sheehy et al., 2012; Bowers et al., 2019).

Even with these best-available video-based eye trackers, it is not straightforward to determine what the source of the fluctuations in the eye movement signal is because the eye movements of interest to fixational eye-movement researchers can have magnitudes close to, or even below, the noise floor of the eye tracker. One may therefore resort to knowledge about the dynamics of physiological movements and measurement noise to characterize the content and possible origin of a gaze position signal (e.g., Findlay 1971; Eizenman et al., 1985; Coey et al., 2012; Bowers et al., 2019). While formal analyses of the dynamics of the gaze position signal usually examine its spectral composition (ibid), we think there is value in training the scientist’s visual pattern recognizer to discriminate between the different types of signals. Here we will therefore first examine a set of example gaze position signals collected from human and artificial eyes, before introducing the analysis techniques that will be used in this paper.

### Oculomotor drift

**Fig. 2:**
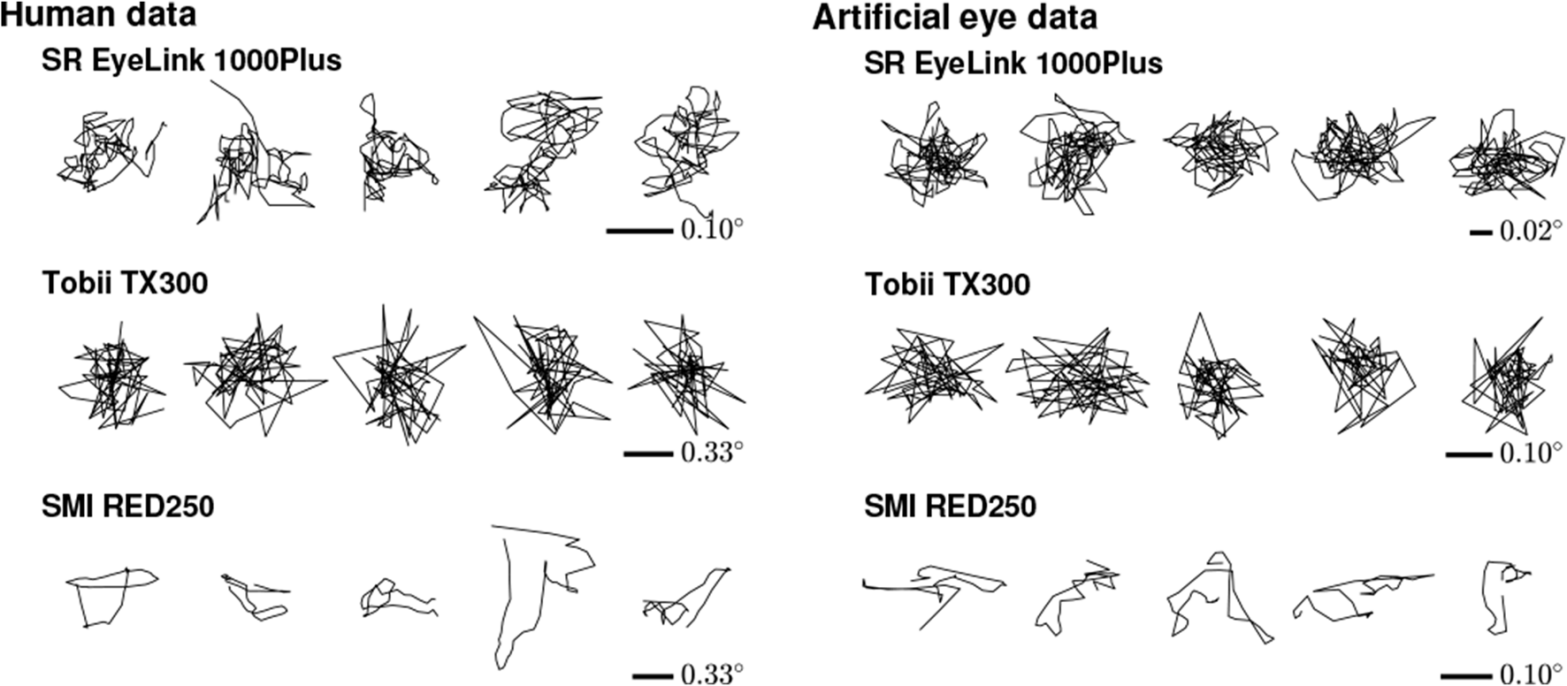
Gaze position data examples. For three trackers, example 200-ms segments from five fixations recorded from humans (*left*) and example 200-ms segments of data recorded with an artificial eye (*right*). Compared to the 300-Hz Tobii data in the middle row, the 1000-Hz EyeLink and 250-Hz SMI data in the top and bottom rows look smoother. Note that the scale at which data is visualized differs for each eye tracker and panel to make differences between the signals easier to see. The scale of the signals is therefore indicated for each eye tracker’s data. Note also that the scale of the data recorded from human eyes is about three times larger than that for the data recorded from artificial eyes

Different signal dynamics are readily seen when examining gaze position data recorded from humans (Fig. 2a) or from artificial eyes (Fig. 2b). While some eye trackers produce data that show large sample-to-sample steps and look essentially randomly distributed around a central point (Tobii TX300), data from other eye trackers (SR EyeLink 1000 Plus and SMI RED250) show smoother trends that appear more similar to a random walk (see also Blignaut & Beelders, 2012, who provide the visual diagnosis that these smooth gaze position signals look like “ant trails”). Since oculomotor drift looks like the smoother signals in Fig. 2 (e.g., Ko et al., 2016; and Engbert et al., 2011), when seeing such signals in the eye tracker with the lowest noise magnitude among the three shown in the plot (the SR EyeLink 1000 Plus), it is tempting to conclude that this eye tracker has a low enough noise magnitude to render oculomotor drift visible in the gaze position signal. That these smooth signals are not visible in an eye tracker with higher noise magnitude, the Tobii TX 300, may be thought to strengthen this conclusion.

However, as Blignaut and Beelders (2012; see also Bedell & Stevenson, 2013) already warned, caution is warranted in drawing such conclusions, as smooth gaze position signals could also be produced by noise suppression systems such as filters in the eye tracker’s software or hardware. This call for cautious interpretation receives further weight from finding such smooth signals also in another tracker than the EyeLink, viz. the SMI RED250. As the SMI RED250’s noise magnitude is at least as large as that of the Tobii TX300, it should be wondered whether the smooth gaze position signals produced by this eye tracker reflect fixational eye movements.

Moreover, as seen in Fig. 2b, these smooth signals also exist in data recorded with static artificial eyes on both the low-noise EyeLink and the higher-noise SMI RED250. During these recordings, we took care to ensure that any physical movement of these artificial eyes relative to the eye tracker (e.g., due to vibrations of the table on which the eye tracker was placed) was likely well below the noise floor of the eye trackers and thus undetectable in the eye trackers’ output. As such, smooth gaze position signals in these recordings could not be due to any physical movement of the tracked artificial eyes. That smooth gaze position signals which appear similar to those recorded from human eyes (if at smaller magnitude) are nonetheless seen in these recordings therefore adds further support to the idea that finding smooth gaze position signals in an eye tracker’s output does not necessarily entail that oculomotor drift is being measured. Instead, the smoothness may be due to filters in the eye tracker. These inconsistencies between eye trackers in whether recorded gaze position signals are smooth or not highlight that it is important to uncover whether the smooth signals reflect true rotations of the eye or whether these signals are artifactually generated by filters in the eye tracker.

### Spectral analyses

As discussed, gaze position signals can range from smooth to spiky in appearance. These smooth and spiky gaze position signal types in fact lie along a continuum that can be described by a single parameter, the spectral color of the signal. The spectral color of a signal is a description of the power in the signal at different temporal frequencies. There is a long history of applying frequency analyses to eye-tracking data in general (e.g., Stark et al., 1961; Campbell et al., 1959; Bahill et al., 1981) and for fixational eye movements in specific (e.g., Findlay, 1971; Eizenman et al., 1985; Stevenson et al., 2010; Coey et al., 2012; Sheehy et al., 2012; Ko et al., 2016; Bowers et al., 2019). Here, we will provide a brief discussion of how the spectral color of gaze position signals is interpreted; for a more detailed discussion of spectral analyses of eye-tracking data, see Niehorster et al., (2020c).

**Fig. 3:**
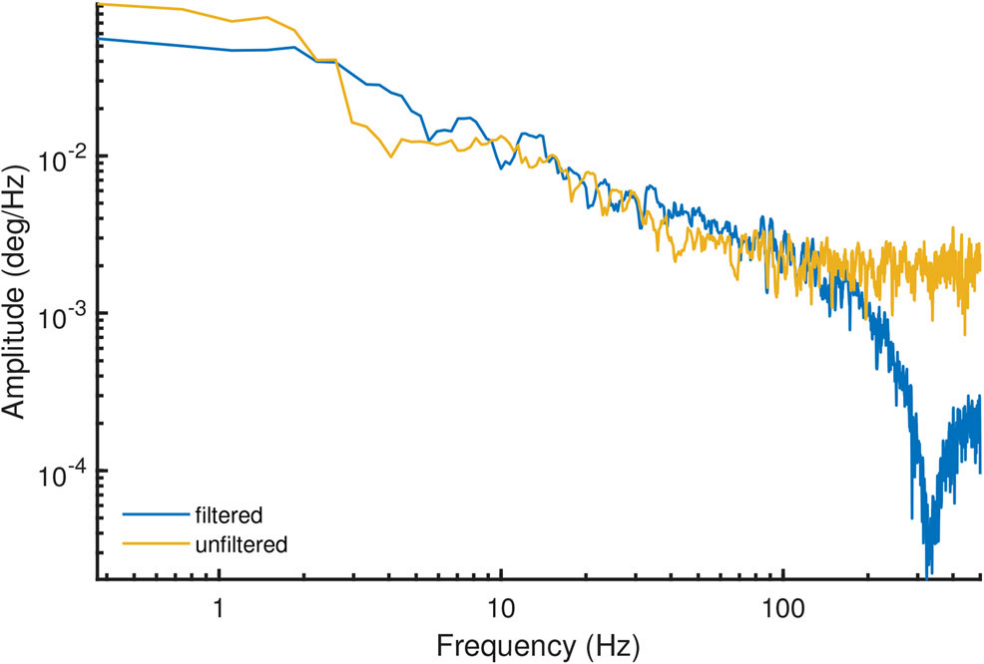
Amplitude spectrum example. Amplitude spectra for the fixational eye movement traces shown in Fig. 1, computed using the multitaper method (Thomson, 1982; Babadi and Brown, 2014). These data were recorded with an SR EyeLink 1000 Plus at 1000 Hz, with its filters either turned on or turned off

As an example, the temporal spectral decomposition of the gaze position signals of Fig. 1 is shown in Fig. 3. Specifically, Fig. 3 shows amplitude spectra, allowing to directly read off the amplitude of gaze movement at a given frequency. As can be seen, for the fixation recorded with the EyeLink’s filter switched off (yellow line), the spectrum appears to consist of two distinct segments, i.e., a segment at the lower frequencies that slopes downward and after approximately 100 Hz, a flattened out segment. Both the slopes in the amplitude spectrum and the location of the flattening-out are consistent with the above-cited literature.

In the literature, the downward-sloping segment is often interpreted as due to fixational eye movements. A signal with a straight-line downward slope as spectrum is said to exhibit 1/*f* dynamics, because the shape of the spectrum is characterized by the equation 1/*f*^*α*^. Here, *f* is frequency and *α* is a scaling exponent reflecting the slope of the line, which characterizes the scaling of the signal’s power with frequency. In this case, the power spectral slope of this segment is close to 6 dB/octave (i.e., 1/*f*^2^), and reflects a signal that looks smooth. Such scaling is what would be expected for the random walk-like nature of ocular drift (Cornsweet, 1956; Findlay, 1971; Burak et al., 2010; Engbert et al., 2011; Nyström et al., in press).

Such signals with non-zero spectral slope are referred to as “colored”. In contrast to this colored segment of the power spectrum stands the flat segment observed at higher frequencies (Fig. 3). Such flat power spectra where signal power is constant over frequency are called white signals, and appear random and more spiky. For eye-tracking signals, such signal dynamics are often attributed to measurement noise. Since fixational eye movements have a bandwidth of up to about 100 Hz (e.g., Findlay, 1971; Ko et al., 2016; Bowers et al., 2019), it is expected that the spectrum at higher frequencies only reflects such measurement noise. Note that if the measurement noise of an eye tracker is too large, it will drown out the 1/*f* component of the gaze position signal that is due to fixational eye movements, indicating that the eye tracker is not sensitive enough to measure the eye movements of interest.

An understanding of the noise characteristics of the measurement device is critical when studying gaze dynamics by means of examinations of signal color because it must be ascertained that the source of the 1/*f* dynamics in the gaze position signals is due to the participant’s eye movements and neither the measurement noise produced by the eye tracker nor a filter in the eye tracker. As can be seen by contrasting the two spectra in Fig. 3, the filters of the EyeLink strongly change the shape of the measurement noise part of the signal’s spectrum (i.e., beyond 100 Hz). Indeed, while measurement noise in an eye tracker is likely white if each output sample is processed independently, 1/*f* power law behavior in the signal can be introduced not only due to the dynamics of a rotating human eye but also by applying temporal filters to the recorded gaze signals. This was, for instance, shown by Coey et al., (2012), who recorded an artificial eye (thus exhibiting no fixational eye movements) with an eye tracker and examined the color of the resulting gaze position signal. They reported that the signal in their ASL eye tracker was indeed white, as would be expected for measurement noise, when the eye tracker’s averaging filter was switched off, while switching on this noise suppression system yielded colored gaze position signals.

Wang et al., (2016) have extended Coey et al.,’s (2012) results to 12 further eye trackers, and report white signals reflecting measurement noise when recording from artificial eyes for each of the systems they examined. These results of Wang et al., (2016), however, appear inconsistent with a report by Blignaut and Beelders (2012), who have examined two eye trackers that were also reported on by Wang et al., (2016). Blignaut and Beelders (2012) found that one of the two eye trackers exhibited the kind of smooth traces that characterize colored signals, even when recording from an artificial eye. Although Blignaut and Beelders (2012) did not perform frequency analyses, their findings suggest that eye trackers may produce gaze position signals that exhibit 1/*f* dynamics even in the absence of any physical eye movement, which is at odds with the findings of Wang et al., (2016), but consistent with those of Coey et al., (2012).

### Aims of this paper

Here we examine whether color (visually identified as smooth gaze position signals) in the output of an eye tracker reflects fixational eye movements or whether these signals are instead due to filters in the eye tracker. For this purpose, the spectral composition of gaze position signals obtained from human and artificial eye recordings made with five video-based eye trackers is analyzed.

We posit the following two models for the origin of colored signal dynamics in eye tracker data. First, Wang et al., (2016) reported that data recorded with artificial eyes are always white, while data recorded from humans are always colored. Based on this observation, they speculated that the color in human eye-tracking data originates from fixational eye movements. We will refer to this statement as the *oculomotor hypothesis*.

The second hypothesis, the *filter hypothesis*, states that the color in eye-tracker data is due to temporal filters in the eye tracker hardware or software. The filter hypothesis offers an explanation for why data recorded from artificial eyes can also appear smooth (cf. Blignaut and Beelders, 2012 and Fig. 2 above) and exhibit color (cf. Coey et al., 2012).

Under these hypotheses, we may predict the following outcomes for our measurements. To generate these predictions, we assume that unfiltered measurement noise is white (see, e.g., Coey et al., 2012; Wang et al., 2016), which is also borne out by the results reported below. Table 1 summarizes the predicted results under the two hypotheses. First, under the oculomotor hypothesis, as reported above, we would predict that all signals recorded from human eyes are colored, while all signals recorded from artificial eyes are white. In contrast, under the filter hypothesis, data recorded with artificial eyes are white at the early processing stages (e.g., determining the location of the pupil and corneal reflection features, as well as gaze estimation), but become colored in later stages of gaze signal processing through the application of filters. In the case of the filter hypothesis, a similarly colored signal is thus expected for data recorded from human and artificial eyes.

**Table 1:** Expected results under the oculomotor and filter hypotheses in terms of signal color as denoted by the signal’s power spectrum slope *α* (white or

	Human data	Artificial eye data
Oculomotor hypothesis		
Filtered		white (*α* = 0)
Unfiltered		white (*α* = 0)
Filter hypothesis		
Filtered		
Unfiltered	white^‡^ (*α* = 0)	white (*α* = 0)

*If the measurement noise magnitude of the eye tracker is large, white signals may be found in human unfiltered data. ^‡^If the measurement noise magnitude of the eye tracker is low enough, some signals may be found in human unfiltered data.

Note, however, that the outcomes of our measurements may not conform strictly to one of these hypotheses. Specifically, if the magnitude of measurement noise of an eye tracker is much larger than that of fixational eye movements, we may expect to find that human unfiltered eye-tracker data is white also under the oculomotor hypothesis. Conversely, under the filter hypothesis, if the measurement noise magnitude of an eye tracker is sufficiently small so as to render some fixational eye movements detectable in its signal, the unfiltered signal can also be expected to exhibit color when recording from a human. Signal color in this case may however reflect not only fixational eye movements but could also arise due to, for instance, incomplete compensation for head movement, or deviations in the gaze position signal caused by changes in pupil size (Wyatt, 2010; Drewes et al., 2012; Drewes et al., 2014; Choe et al., 2016; Hooge et al., 2019).

Some of this material has previously been presented in Holmqvist and Andersson (2017 pp. 179–182). Furthermore, the data analyzed in this paper are also used for parallel analyses in Niehorster et al., (2020c), which provides an overview of various measures for characterizing eye-tracking signals, and investigates how these measures relate to the slope of the signal’s power spectrum *α* used in this paper.

## Method

### Participants and artificial eye

Human data were acquired from three volunteers and author DN, yielding data from a total of eight eyes. One participant wore glasses, three did not. The participants provided informed consent.

We also recorded data from a set of artificial eyes provided by SMI GmbH. The same set was previously used by Wang et al., (2016).

### Apparatus

Gaze position signals were recorded on five eye trackers: the SR Research EyeLink 1000 Plus in desktop mount and head stabilized mode at 1000 Hz, the SMI RED250 at 250 Hz, the SMI RED-m at 120 Hz, the Tobii TX300 at 300 Hz, and the Tobii X2-60 at 60 Hz. Recordings were performed with the default settings of the eye trackers, i.e., any filters were left on if they would be on by default. Recordings with the EyeLink were controlled by the EyeLink Toolbox (Cornelissen, Peters & Palmer, 2002), the SMIs with an early version of SMITE (Niehorster & Nyström, 2020b) and the Tobiis with an early version of Titta (Niehorster, Andersson & Nyström, 2020a). For the EyeLink 1000 Plus, we have made additional recordings with its heuristic filter switched off. For the SMI RED-m, the default setting to average the gaze position data for the two eyes was switched off to yield separate signals for each eye. Viewing distance was approximately 65 cm. Participants were unconstrained, except for the Eyelink, where chin and forehead rests were used.

### Stimuli and procedure

**Fig. 4:**
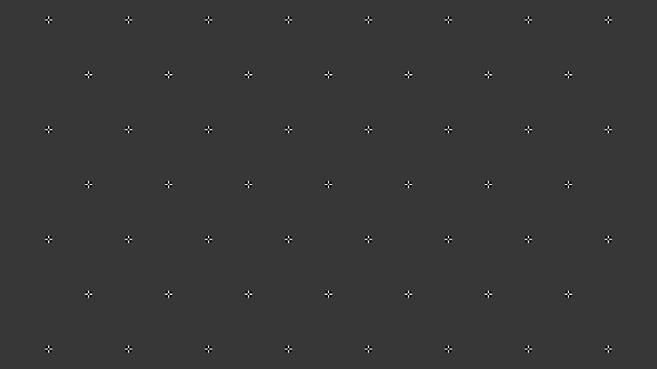
Fixation target locations. The 53 fixation targets were laid out on a 4 x 8 rectangular grid, with another 3 x 7 rectangular grid placed on top such that its points coincided with the centers of the cells formed by the 4 x 8 grid.

After a default calibration (i.e., the default number of calibration points for the system in their default configuration) as was appropriate for the specific eye tracker and setup, we had participants look at a further series of points on the monitor. These points (a 1.6-cm-wide black circle overlaid with a white cross and a 0.3-cm-wide centered black circle, following Thaler et al., 2013) were distributed in a 4 x 8 rectangular grid. Further points were placed on a 3 x 7 rectangular grid such that they were at the centers of the cells defined by the 4 x 8 grid. This leads to a total of 53 points (see Fig. 4). To avoid differences between eye trackers due to the different screen sizes used, for all eye trackers this grid spanned (horizontally and vertically) 45.5 x 26.8 cm. After fixating the center of the screen, the fixation points were presented for 1500 ms each in a random sequence containing each location four times, yielding a total of 213 presented points in a session.

For recordings with artificial eyes, an experimenter first calibrated the eye tracker. The pair of artificial eyes, mounted on a heavy tripod at 6-cm separation from each other, was then positioned at the location where the experimenter’s eyes were during calibration. After a brief resting period, data recording was then started and ran for 19 s while the experimenter left the room. This procedure followed established practice in the field (e.g., Coey et al., 2012; Wang et al., 2016; Holmqvist and Blignaut, 2020) and was required because not all the eye trackers would deliver data without a prior calibration, and we did not have a way to perform a calibration using the artificial eyes themselves.

### Analysis

#### Window selection

To test our hypothesis, we had to compute the slope of the power spectrum of the gaze position signal during each fixation. To be able to compute this for each fixation point, we first had to select a time window of data points to analyze. To do so, we developed a window selection method that aims to place the window we take data from as close to the presented fixation point as possible. Ideally, the method should not rely on any measure of fixational stability so as not to bias the signal’s spectrum. It should also not rely on a fixation classification algorithm, as we could not guarantee that any of the known algorithms is sufficiently reliable in producing comparable windows across the large range of sampling frequencies and noise magnitudes of the eye trackers employed in this examination. The procedure to select a data window for each presented fixation point was as follows. A 200-ms window (Hooge et al., 2017) slid over a section of data ranging from 200 ms after fixation point onset until fixation point offset. To exclude windows that likely contained a (micro-)saccade, we then performed the following procedure. For each possible window position, the dispersion of the samples in the window,

1 $$ \sqrt{\left( \max(x)-\min(x)\right)^{2}+\left( \max(y)-\min(y)\right)^{2}} $$

was calculated. For each fixation, we then excluded half of the candidate window positions, i.e., those that yielded the 50% largest dispersion values, from further consideration. For the remaining window positions, the average gaze position during the window was calculated. The window for which the average gaze position was closest in space to the fixation point was selected as the window for which the spectrum was analyzed. For the data recorded from the artificial eyes, measures were calculated for a 200-ms window that was moved across the entire recording in 50-ms steps.

As we are not interested in the eye movements of the participant but in the gaze position signals, we treated each eye independently. The below analyses thus report results for eight eyes. “Eye” in the below text refers to one of these unique eyes. The above window selection method was executed separately for the data from each eye. No differences between data from the participants’ dominant and non-dominant eyes were found (all *p* values of dependent samples *t* tests > 0.91).

#### Amplitude spectra

Amplitude spectra for the gaze position signals of each eye were separately computed for the horizontal and vertical channels using the function periodogram from the MATLAB (Natick, MA, USA) Signal Processing Toolbox, with the default rectangular window. The output of this function is a power spectrum. To create amplitude spectra, the square root of the power spectra was computed.

The slope of the power spectrum (scaling exponent *α*) was determined by fitting a line in log-log space to the power spectrum using the MATLAB function *polyfit*. Note that although slightly uneven inter-sample intervals were reported in the data for some of the eye trackers, possibly due to variations in camera framerate or jitter in software timestamps (standard deviations of the human data’s inter-sample interval [ISI] as reported by the eye trackers were 0, 0.74, 9.98, 1.15, and 1.89% of the nominal ISI of, respectively, the SR EyeLink 1000 Plus, SMI RED250, SMI RED-m, Tobii TX300, and Tobii X2-60), we found the same results when using the Lomb–Scargle periodogram method that Wang et al., (2016) recommended be used for unevenly sampled data. Estimates of *α* calculated from these two periodogram methods correlated very highly, *R*^2^ > 0.99.

#### Unfiltered data

Where possible, the analyses in this paper were done both for gaze position signals recorded with the eye tracker’s default filters applied, and for data that were recorded with any configurable filters switched off. For the EyeLink, besides the set of recordings with its heuristic filter set to its default level (2, “extra”), we also made recordings with the heuristic filter switched off for a subset of participants.

The SMI eye trackers tested do not offer the option to switch off the filters applied to the gaze position signal that is provided in screen coordinates. Both SMI eye trackers, however, also provide a gaze vector in SMI’s headbox coordinate system in their data files, and we suspect that no temporal filters are applied to these data during gaze estimation (our analyses below corroborate this assumption). To enable analyses of unfiltered SMI data, we therefore decomposed the gaze vectors for each eye into Fick angles (Fick, 1854; Haslwanter, 1995), and then applied the methods described above to calculate the periodogram and power spectrum slope from the resulting eye orientation data.

Tobii claims in their product documentation that the TX300 and X2-60 do not apply any temporal filter to the recorded gaze position signals and that these machines thus always deliver unfiltered data. Our analyses below indeed appear to confirm this claim. As such, for recordings made with the Tobii eye trackers, we only present analyses of unfiltered data.

## Results

In this result section, we examine plots of the amplitude spectra of the recorded data. Along with these figures, we have also listed the corresponding slopes of the power spectra (scaling exponent *α*) for the five eye trackers in Table 2. Scaling exponents for both human data and data recorded from artificial eyes are listed both with the eye tracker’s filters switched on where possible, and with the filters switched off. Furthermore, Table 2 lists both scaling exponents derived by fitting lines to the entire frequency range of the power spectrum, and scaling exponents for fits to only the first 100 Hz of the power spectrum. The latter fits indicate gaze position signal dynamics in the frequency range of fixational eye movements (see above).

**Table 2:** Power spectrum scaling exponents (*α*) for filtered and unfiltered human and artificial eye data determined by fitting a line to (*left columns*) the entire power spectrum, and (*right columns*) the first 100 Hz of the power spectrum

Eye tracker	Fit to entire power spectrum				Fit to first 100 Hz			
	Filtered		Unfiltered		Filtered		Unfiltered	
	Human	Artificial eye	Human	Artificial eye	Human	Artificial eye	Human	Artificial eye
EyeLink	2.281	2.328	0.544	0.081	1.307	0.466	1.179	0.219
RED250	2.280	2.456	0.903	–0.001	2.353	2.482	1.004	0.006
RED-m	0.886	1.364	0.330	0.210	0.886	1.364	0.330	0.210
TX300	–	–	0.280	0.182	–	–	0.351	0.224
X2-60	–	–	0.382	0.308	–	–	0.382	0.308

The power spectrum scaling exponents presented in this table were derived by averaging the exponents for horizontal and vertical gaze position data. Note that for the Tobii eye trackers, only unfiltered data were available. Note also that as the SMI RED-m and Tobii X2-60 had sampling frequencies below 200 Hz, fits to their entire spectrum were used for all entries in the table.

### Data at default settings

**Fig. 5:**
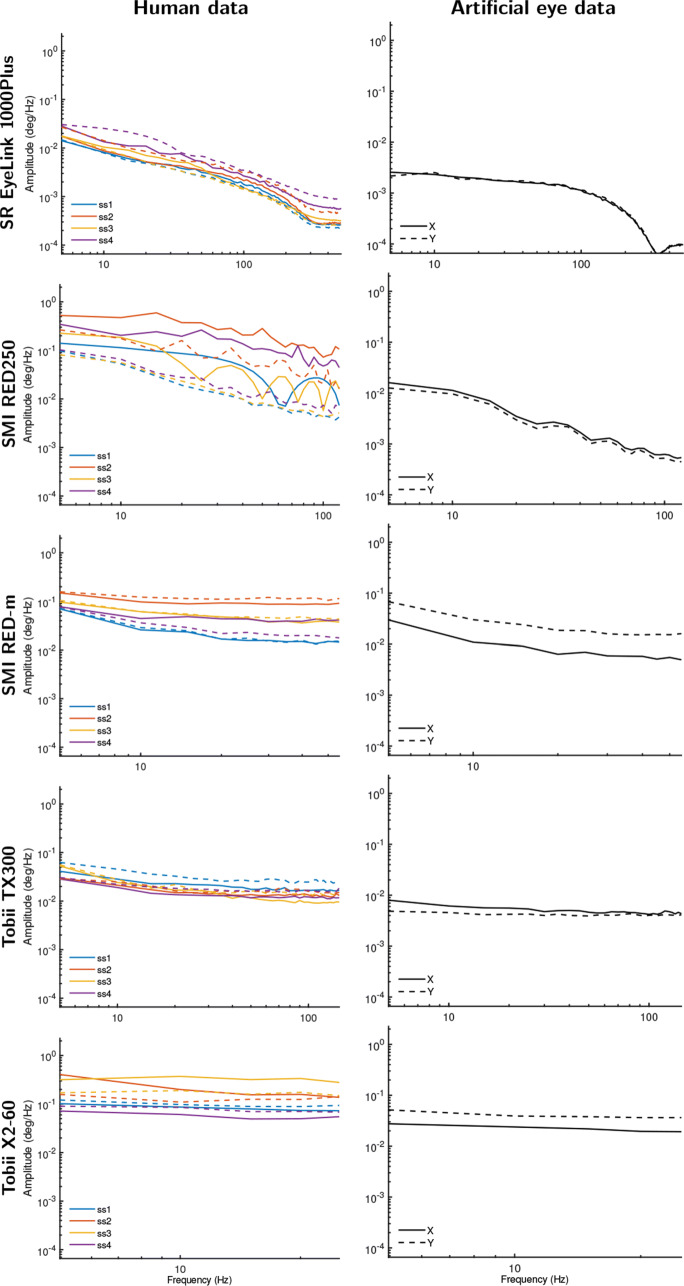
Amplitude spectrum plots for data recorded at each eye tracker’s default settings from human eyes (*left column*) and artificial eyes (*right column*). *Different color lines* denote different participants. *Solid lines* show amplitude spectra derived from horizontal gaze position data, and *dotted lines* for vertical gaze position data

Figure 5 shows amplitude spectra derived from gaze position data from the five eye trackers, with human data presented in the left column and data recorded from artificial eyes in the right column. First we examine the two Tobiis, the TX300, and the X2-60. Note that both eye trackers only provided unfiltered data, and as such amplitude spectra for unfiltered data are plotted in Fig. 5. As can be immediately seen, data for both eye trackers showed amplitude spectra that are close to flat, corresponding to scaling exponents that were close to zero (Table 2). This indicates that their signals are close to white, for both artificial eyes and human data. Importantly, the spectral slope is the same for both human and artificial eyes for both eye trackers, even though the magnitude of variability in the signal—the height of the amplitude spectrum in the plots—is larger for human data than for artificial eye data. This finding does not have a bearing on the oculomotor vs. filter hypothesis discussion because we cannot exclude the possibility that these two eye trackers produced a white measurement noise component that was sufficiently large when recording from human eyes to drown out possible small fixational eye movements that may otherwise have been recorded.

At their default setting of providing filtered data, the SMI eye trackers exhibited a different type of signal than the two Tobiis. The amplitude spectra for data from the RED-m, and especially the RED250, showed a clear downward slope. This corresponded to scaling exponents for filtered data that were around 1 and 2, for the RED-m and RED250, respectively, for both human data and data recorded from artificial eyes. This indicates that both eye trackers provide gaze position signals with significant color and replicates the report of Blignaut and Beelders (2012) that an SMI RED250 produces smooth data when recording from an artificial eye (see also Fig. 2), but is inconsistent with the report of Wang et al., (2016) that both these SMIs produced white signals in this case. These data offer a strong contradiction to the oculomotor hypothesis because the data are not consistent with the expectation derived from this hypothesis that recording from artificial eyes should yield white signals.

Finally, the EyeLink 1000 Plus, like the two SMIs, exhibits a downward slope in the amplitude spectrum, although at a much lower magnitude than the SMIs. As was the case for the other four eye trackers, for the EyeLink, the human data were noisier than the artificial eye data, but not qualitatively different. When its filters were switched on, the EyeLink’s data yielded colored signals both when recording from human eyes and when recording from artificial eyes. As was the case for the data of the SMI eye trackers, this finding is in contradiction to the oculomotor hypothesis, and is inconsistent with the white signals reported by Wang et al., (2016) for an EyeLink when recording from artificial eyes.

However, while the scaling exponent for the EyeLink data was very similar for human and artificial eye data when it was determined from the entire power spectrum (see Table 2, left columns), the scaling exponent was much larger for the human data than for the artificial eye data if it is computed for data only up to 100 Hz (see Table 2, right columns)—the frequency range of fixational eye movements. This indicates that the EyeLink’s filter has a larger effect in the frequency range beyond 100 Hz than for the first 100 Hz, and interestingly also suggests that likely at least part of the observed color in the human data is due to fixational eye movements or artifacts such as the slow-varying deviations in recorded gaze position caused by fluctuations in pupil size (Wyatt, 2010; Hooge et al., 2019), and not only due to the EyeLink’s filter. This finding may therefore suggest that the noise magnitude in the EyeLink recordings was low enough to enable recording some fixational eye movements. Note that the scaling exponents were very similar for the two frequency ranges for all the other eye trackers.

In summary, the above analysis shows that for each eye tracker, the color of the signal varied little between data recorded from human and artificial eyes. This pattern of findings across the five eye trackers is inconsistent with the hypothesis that fixational eye movements are the origin of colored signals in eye-tracker recordings. If this were the case, we would have instead expected to see different signal colors in human and artificial eye data for most eye trackers and, importantly, that data recorded from artificial eyes would consistently have exhibited white signals.

### Unfiltered data

The observations reported above are consistent with the filter hypothesis, which states that the color of the signal and hence the smoothness of its visual appearance derive from filters applied by the eye tracker. If such filters are the predominant cause of the colored dynamics observed in video-based eye-tracker data, it would be expected that the signal color is similar for both artificial eyes and human data for each eye tracker, which is what we observed. Importantly, it would furthermore be expected that the signal would appear white when such filters are switched off or when data from an unfiltered stage of the eye tracker’s gaze estimation pipeline are examined. In order to provide this further test of the filter hypothesis, next we report on unfiltered data acquired with the EyeLink and SMI systems.

For the EyeLink 1000 Plus, new recordings were made with an artificial eye and for two of the participants using an identical setup and procedure as the previous recordings, but with its heuristic filter switched off.

**Fig. 6:**
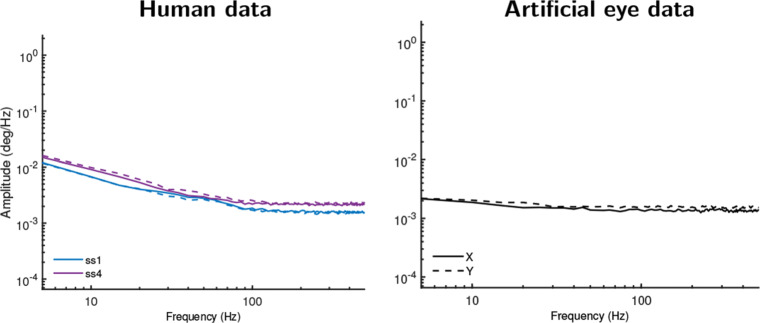
Amplitude spectra for unfiltered EyeLink 1000 Plus data. Recordings made with SR EyeLink 1000 Plus with its heuristic filter turned off and recording from human eyes (*left column*) and artificial eyes (*right column*). Artificial eye data now exhibit a flat amplitude spectrum (white signal). The slope for data recorded from humans has become much shallower than when recording with the filter switched on, as shown in Fig. 5, but still exhibits some color. *Different color lines* denote different participants. *Solid lines* show amplitude spectra derived from horizontal gaze position data, and *dotted lines* for vertical gaze position data

Figure 6 presents amplitude spectra of these data, which can be compared to the top row of Fig. 5 where recordings made with the filter switched on are presented. As Fig. 6b clearly shows, unfiltered EyeLink data recorded with an artificial eye yielded a white signal, in stark contrast to the colored signal observed when recording with the filter switched on. Furthermore, in Fig. 6a, it is seen that the unfiltered human data were also much whiter (flatter slope) than when recording with the filter switched on. This shows that the color of the signal recorded from human eyes with the EyeLink was for an important part due to its heuristic filter, as expected under the filter hypothesis. Closer examination of Fig. 6a and comparison with Fig. 5 however allows us to add some nuance to this conclusion. In this comparison, it is seen that the scaling exponents for filtered and unfiltered human data when determined over the first 100 Hz (cf. Table 2) of the signal were nearly identical. This suggests that the EyeLink’s heuristic filter essentially acts as a low-pass filter, and has only minimal effect in the frequency range that contains information about fixational eye movements, but sharply colors the frequency range beyond it.

The slight color that remains in Fig. 6a in the human gaze position signals recorded with the EyeLink after turning off the heuristic filter is not indicative of further, hidden filters, because such filters would also have colored the signal recorded with artificial eyes. We cannot rule out that the colored signal dynamics may reflect deviations in the gaze position data due to uncompensated head movements, but consider this unlikely since the recordings with the EyeLink were made on experienced participants whose heads were stabilized on chin and forehead rests, which would minimize head movements. Instead, the remaining signal color indicates that the EyeLink’s measurement noise magnitude is low enough that it may in fact pick up some fixational eye movements. If so, these constitute mainly drift as our analysis-window selection procedure should have excluded most segments that contained microsaccades. The significant color in unfiltered human EyeLink data seen in Fig. 6a when examining the first 100 Hz supports this interpretation. Alternatively, however, the remaining color may reflect the slow-varying deviations that artefactually occur in the recorded gaze position of video-based eye trackers due to fluctuations in pupil size (e.g., Wyatt 2010; Hooge et al., 2019).

**Fig. 7:**
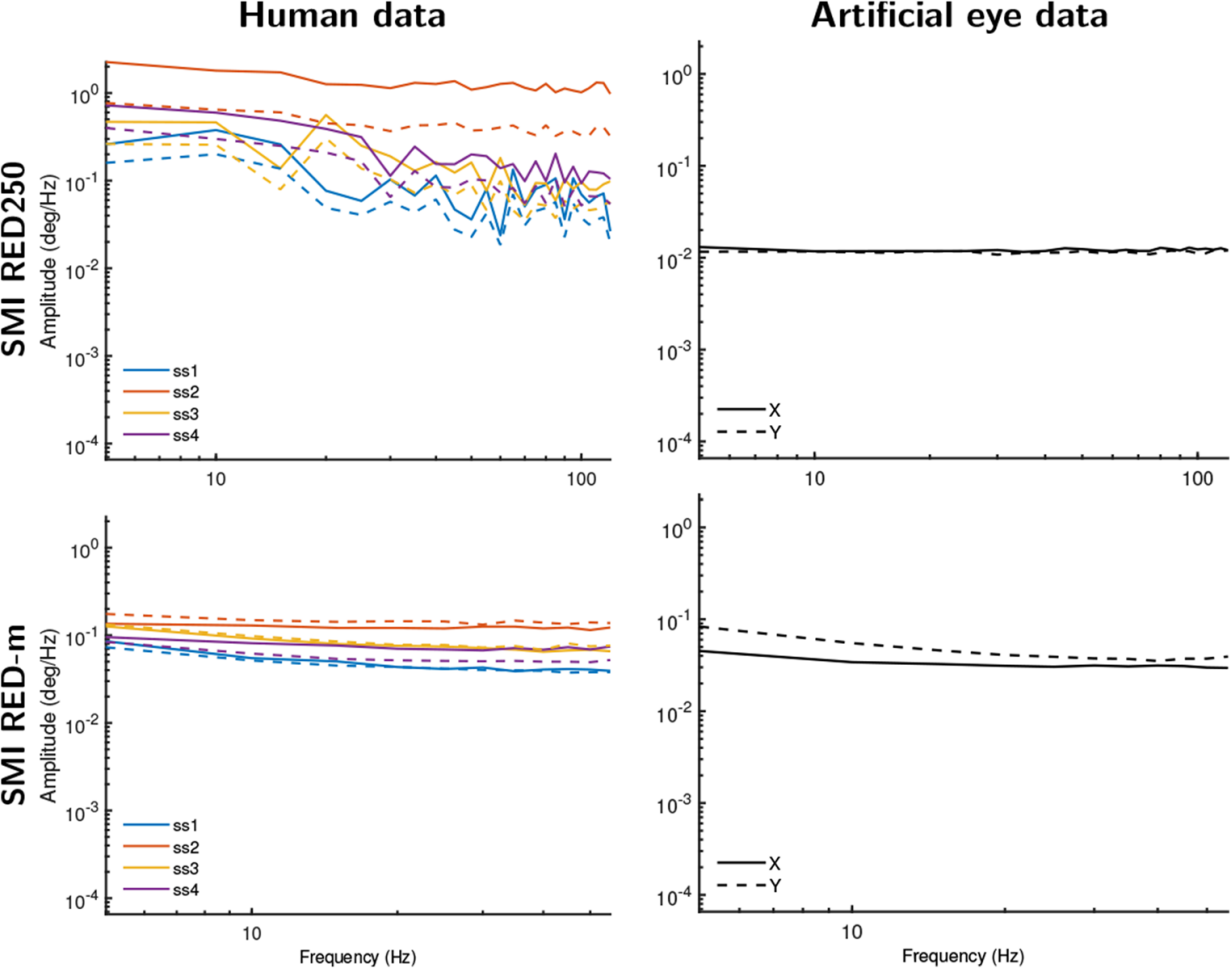
Amplitude spectra for gaze vector data from the SMI eye trackers. As not all filters could be turned off in the SMI RED systems, we instead analyzed the gaze vector data provided by these eye trackers for both human eyes (*left column*) and artificial eyes (*right column*). Except for the human data recorded on the SMI RED250, all gaze vector data exhibit white signal dynamics. The human gaze vector data on the SMI RED250 nonetheless exhibits amplitude spectra that are much less colored than the filtered gaze position data shown in Fig. 5. *Different color lines* denote different participants. *Solid lines* show amplitude spectra derived from horizontal gaze position data, and *dotted lines* for vertical gaze position data

For the SMI eye trackers, it was not possible to turn off all filters, but the data files include gaze vector information that is an intermediate representation of gaze used to determine gaze position on the screen (see “Unfiltered data” in the “Method” section above). We posited that these gaze vectors may have undergone less filtering than the gaze position data. Figure 7 plots amplitude spectra based on the data derived from these gaze vectors from the same SMI RED-m and RED250 recordings as presented in Fig. 5. Indeed, for all data except the human SMI RED250 data, the data are now practically white signals, consistent with the expectation that the gaze vectors in these SMI systems are unfiltered, and importantly that the color and smooth appearance of data from these systems is due to filtering of the gaze position signal provided by these eye trackers. The human SMI RED250 gaze vector data still exhibit signals that are clearly more white than the corresponding filtered human and artificial eye gaze position data. That human gaze data on the SMI RED250 retains some color when bypassing its filters suggests that imperfect head-movement compensation may have caused deviations in the data (participants were not stabilized on a chin rest in this setup, which may cause such deviations, cf., Niehorster et al., 2018), may be due to the pupil-size artifact in gaze data, or similar as for the EyeLink above indicates that the SMI RED250 can measure human oculomotor drift. We consider the latter explanation to be very unlikely given that the RED250’s noise magnitude is almost an order of magnitude larger than that of the EyeLink, which would likely render any fixational eye movements undetectable.

In summary, these further analyses confirm that the smooth colored signal characteristics seen in artificial eye data recorded with the EyeLink and SMI systems (cf. Fig. 5) are due to filters in these eye trackers. For human data, the results furthermore strongly suggest that filters, and not fixational eye movements, are the main cause of the color observed in the gaze position signals provided by these eye trackers.

## Discussion

In this paper, we have reported analyses of the spectral color observed in gaze position signals recorded with video-based eye trackers during human fixation episodes and in data from artificial eyes. Using these data, we examined whether color in the gaze position signal is likely due to fixational eye movements of the human participants, or is instead mostly caused by filters in the eye tracker. Below we discuss the findings of this investigation, and their implications for research into fixational eye movements.

### Eye-tracking data signal properties and their origin

The findings of this paper support the hypothesis that filters, and not fixational eye movements, are the main cause of the color that is frequently observed in the gaze position data of many video-based eye trackers. The oculomotor hypothesis holds that colored (smooth-looking) signals would be observed only in data recorded from human eyes, while data recorded from artificial eyes would be exclusively white (random). Ascribing to this theory, previous work (Wang et al., 2016) has interpreted the pink color they observed in the gaze position data they recorded from human subjects to be of an oculomotor origin, i.e., microsaccades and drift. In contrast, our recordings show that for each eye tracker except the EyeLink, data recorded from human eyes exhibit the same signal color as data from artificial eyes. Even for the EyeLink, whether its heuristic filter was enabled or not caused a much larger change in the color of the recorded gaze position signals than the difference in color between recordings made from human and artificial eyes (contrast the top panels of Fig. 5 with Fig. 6).

As such, across the eye trackers examined in this study, the main contributor to whether the gaze position signal exhibited color was found to be whether filters were applied. This result is consistent with a finding reported by Coey et al., (2012) that data recorded with an artificial eye were white when their eye tracker’s averaging filter was switched off, and colored when it was switched on. Our results are, however, inconsistent with those of Wang et al., (2016), who reported that recording from artificial eyes yielded white signals for all of the eye trackers they examined. As we have discussed and shown in this paper, the filters found in the eye trackers we examined (the SMIs and the EyeLink) necessarily introduce color in the recorded signal. Since it is, to the best of our knowledge, impossible to switch off the filters applied to the gaze position output of the SMI RED250 and RED-m, we find it remarkable that Wang et al., (2016) report that these SMI eye trackers produce white signals when recording from an artificial eye^1^. Our finding of colored signals in the SMI RED250 is furthermore consistent with Blignaut and Beelders (2012), who have previously reported that the SMI RED250 produces smooth-looking (colored) gaze position signals when recording from an artificial eye.

### Implications for fixational eye-movement research

Our results may have important implications for fixational eye movement research. First, when viewing visualizations of recorded eye-tracking data like those presented in the top and bottom panels of Fig. 2a, it is tempting to interpret the smoothly changing signal as being indicative of oculomotor drift, since it looks like and is statistically similar to a random walk. Our results, however, should lead to caution in doing so, since we found that the same kinds of smoothly changing signals can be created by filtering a white signal (see also, Niehorster et al., 2020c). Just like a cautious eye-movement researcher takes care to not infer why participants, for instance, look in a certain order to specific points on a stimulus display unless their research design enables them to do so (e.g., Ballard et al., 1995), care should also be taken to not read too much into the smoothly changing eye movement signal.

Second, for the SMI RED250 and RED-m and the Tobii TX300 and X2-60 eye trackers used in this paper, the authors deem it very unlikely that their data contain a recoverable trace of fixational eye movements. Even though some of these systems provide colored gaze position signals suggestive of ocular drift, when examining unfiltered data from these eye trackers, a white signal with a power spectral density slope close to 0 was found. This suggests that the noise level in these systems is too high to be able to resolve fixational eye movements. Although to the best of our knowledge these four systems have not been used for fixational eye movement research, our results do provide a cautionary tale by highlighting that it is imperative when doing such studies to ensure that the smooth-looking colored gaze position signals output by the eye tracker are not created by a filter that is applied to the eye-tracker data. We further discuss the EyeLink in the Section “The EyeLink” below.

Third, important open questions regarding the use of filters in the recording of eye movements remain, especially as influential work using video-based eye trackers to study fixational eye movements (e.g., Roberts et al. 2013; Engbert and Kliegl, 2004; Engbert et al., 2011; Liang et al., 2005) was performed with the EyeLink’s heuristic filter enabled (Engbert, pers. comm.; Roberts, pers. comm.).^2^ To be able to fully evaluate the work in this field, it is important to establish to what extent filters such as the EyeLink’s heuristic filter lift the signal of interest from the background noise, and to what extent the applied filters instead alter or even create the signal dynamics of interest. Such an effort would especially be of interest since others have noted that the displacements and eye velocities characterizing fixational eye movements are of similar magnitude as the noise in video-based eye trackers such as the EyeLink (Collewijn & Kowler, 2008), which led them to question their suitability for research into fixational eye movements. We furthermore call on authors to explicitly state in their papers which filters were enabled during the gaze-data recordings, and not only report the filters that were used during data analysis.

### Filters in eye trackers

Since filters have an important impact on the output of an eye tracker, is it possible to recognize their presence from an eye-tracker’s data? Luckily, this is a non-issue for the systems of the three eye-tracker manufacturers we examined, as they state in their data exports or communication with customers whether the gaze position signals provided by their system are filtered or not, even if they do not make available the exact implementation of their filters.

It should be noted that the signal type (i.e., whether a signal is white or colored) can be assessed with both the scaling exponent *α* as done in this paper, or even more straightforwardly using new measures introduced in the companion paper, (Niehorster et al., 2020c).^3^ However, these values by themselves do not provide sufficient information to determine whether the recorded data from the eye tracker is filtered. This is because multiple factors can lead to colored signals, e.g., it can (1) be due to the application of filters; (2) be due to pupil-size or participant-movement artefacts; and (3) arise because the eye tracker’s noise magnitude is low enough that fixational eye-movements are represented in the gaze position data. In this study, one of the latter two possibilities was likely the cause of some of the coloring in the gaze position signal recorded with the EyeLink system.

When using systems that are too noisy to record fixational eye movements or when recording from perfectly stabilized eyes, can examinations of the signal’s dynamics detect the presence of all types of filters? No, such analysis only reflects the presence of temporal (anti-)correlations in the assessed signal and as such is not sensitive to filters that do not affect the temporal correlation structure of a signal. This means that, for instance, the downsampling operation done by the EyeLink 1000 and 1000 Plus when outputting gaze position signals at 500 Hz or 250 Hz (i.e., recording at 1000 Hz internally, splitting the signal up in chunks of two or four samples and then averaging each chunk independently)^4^ cannot be detected, because this operation does not introduce temporal dependencies between adjacent samples into the output signal. Furthermore, any other operation on the signal that does not take the signal’s history into account cannot be detected by this analysis technique.

### The EyeLink

For the EyeLink, our results have consistently suggested that its noise magnitude is low enough that it may be possible to record fixational eye movements (drift) with this device. Most telling is that the power spectral density of unfiltered EyeLink data has a significant slope up to 100 Hz (Fig. 6a, Table 2), which suggests that the recorded signal may be of biological origin (e.g., Findlay, 1971). However, caution in making this interpretation is required, because it is possible that the 1/*f*^*α*^ characteristics of the signal output by the eye tracker originate from other sources than physical eyeball rotation. A possible alternative cause is the artefactual changes of the recorded gaze direction due to continuous changes in pupil size (Wyatt, 2010; Drewes et al., 2012; Drewes et al., 2014; Choe et al., 2016; Hooge et al., 2019). This artifact causes deviations in the gaze position signal that can be up to several degrees in size, which is an order of magnitude larger than ocular drift is thought to be (e.g., Ko et al., 2016), and may thus be resolvable above the system noise ceiling much more easily. The amplitude spectra of the EyeLink’s pupil size data (available by running the analyses placed online at https://github.com/dcnieho/FixationalNoise_data) were qualitatively similar to those reported in Figs. 5 and 6, lending some support to this idea. It is, however, possible to use calibration procedures to reduce the effect of pupil size changes on the gaze position signal (Merchant et al., 1974; Drewes et al., 2012). Future research could employ such techniques to resolve whether artefacts due to changing pupil size are an important driver of the intrafixational drift movements recorded with the EyeLink.

## Footnotes

1 For the EyeLink the filter can be switched off, but it is unclear from the method reported by Wang et al., (2016) whether they have done so.

2 It should be noted that in these works from both the Engbert and the Roberts labs, heuristic filter level 1 was used, which is a lower level than the default level 2 that was used in the recordings for the current study and may thus be expected to influence the recorded signal less. Furthermore, most studies from the Engbert lab used an EyeLink 2, which may produce signals with different noise levels and dynamics than the EyeLink 1000 Plus used for the current study.

3 An analysis of the data in this paper that uses these new measures can be generated using the data and analysis scripts that we have made public at https://github.com/dcnieho/FixationalNoise_data. This alternative analysis supports the same conclusion as the analysis presented in this paper.

4 The Tobii Spectrum, at the time of writing, also performs such chunk-based averaging to achieve output sample rates of 300 Hz or 150 Hz while running at 600 Hz internally.
